# Electron plasma diagnostics in ELTRAP by electron cyclotron resonance heating method

**DOI:** 10.1371/journal.pone.0296845

**Published:** 2024-04-18

**Authors:** Faisal Khan, Muhammad Ikram, Mostafa Rashdan, Fahmi Elsayed, Pervaiz Ahmad, Mayeen Uddin Khandaker

**Affiliations:** 1 Department of Physics, Hazara University, Mansehra, Pakistan; 2 College of Engineering and Technology, American University of the Middle East, Egaila, Kuwait; 3 Department of Physics, University of Azad Jammu and Kashmir, Muzaffarabad, Pakistan; 4 Centre for Applied Physics and Radiation Technologies, School of Engineering and Technology, Sunway University, Bandar Sunway, Selangor, Malaysia; 5 Department of General Educational Development, Faculty of Science and Information Technology, Daffodil International University, Dhaka, Bangladesh; The British University in Egypt, EGYPT

## Abstract

Electron cyclotron resonance heating method of Particle-in-Cell code was used to analyze heating phenomena, axial kinetic energy, and self-consistent electric field of confined electron plasma in ELTRAP device by hydrogen and helium background gases. The electromagnetic simulations were performed at a constant power of 3.8 V for different RF drives (0.5 GHz– 8 GHz), as well as for 1 GHz constant frequency at these varying amplitudes (1 V—3.8 V). The impacts of axial and radial temperatures were found maximum at 1.8 V and 5 GHz as compared to other amplitudes and frequencies for both background gases. These effects are higher at varying radio frequencies due to more ionization and secondary electrons production and maximum recorded radial temperature for hydrogen background gas was 170.41 eV. The axial kinetic energy impacts were found more effective in the outer radial part (between 0.03 and 0.04 meters) of the ELTRAP device due to applied VRF through C_8_ electrode. The self-consistent electric field was found higher for helium background gas at 5 GHz RF than other amplitudes and radio frequencies. The excitation and ionization rates were found to be higher along the radial direction (r-axis) than the axial direction (z-axis) in helium background gas as compared to hydrogen background gas. The current studies are advantageous for nuclear physics applications, beam physics, microelectronics, coherent radiation devices and also in magnetrons.

## 1. Introduction

**Fig 1:**
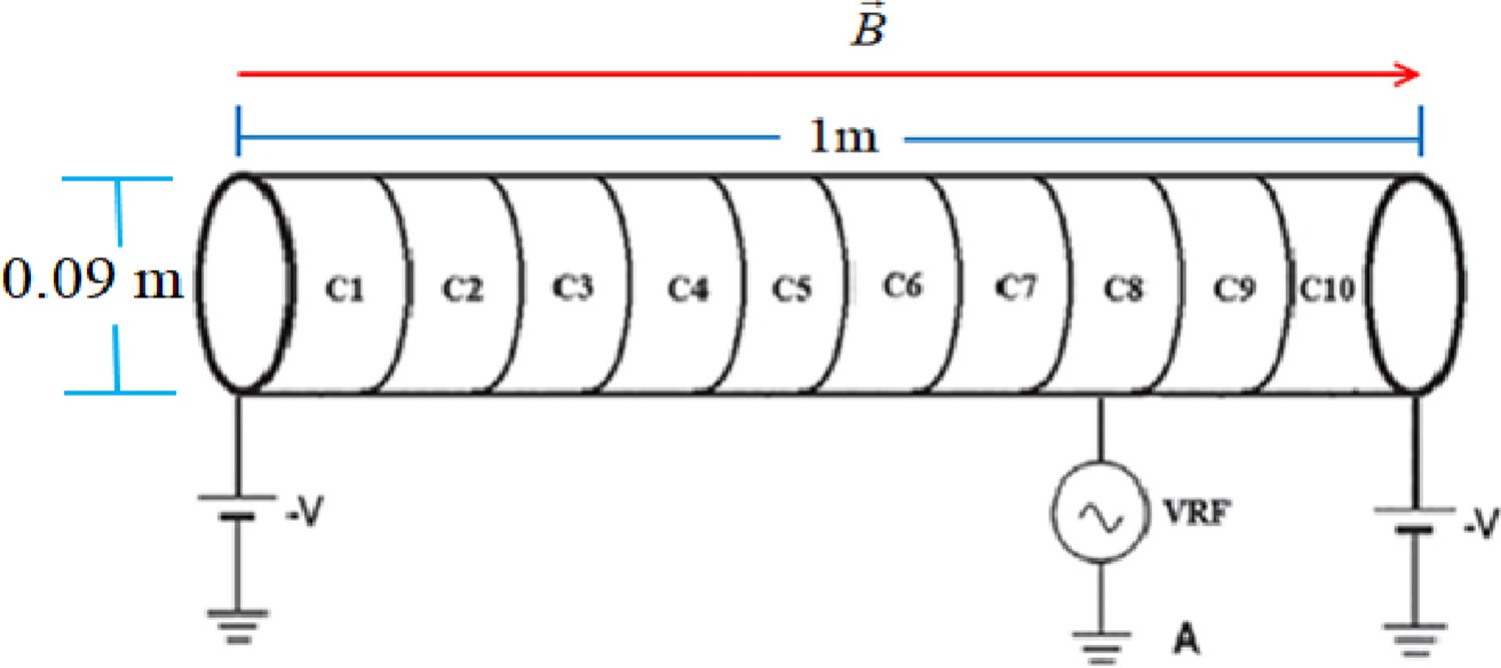
Schematic diagram of ELTRAP for confinement of electron plasma.

Electromagnetic simulation has made significant advancements in confining and understanding both neutral and non-neutral plasmas. The Malmberg Penning trap can be used to confine non-neutral or single-component plasma computationally and experimentally [1]. It consists of a stack of hollow metal cylinders immersed in a magnetic field and is used to study electron plasma dynamics under various conditions. The trap is divided into three sections, with the center being grounded and, on the ends, electric power is applied as shown in Fig 1. There are various diagnostics techniques used to study the characteristics of non-neutral plasma, but we choose the electron cyclotron resonance heating (ECRH) method [2]. This method has made significant contributions in validating heating phenomena in high-density and low-temperature non-neutral plasma in magnetic confinement devices, such as ELTRAP [2, 3]. This technique has gained popularity due to its versatility, easy confinement of electron plasma in ELTRAP devices for a long period, and wide range of applications. In thermal equilibrium state cylindrical symmetry is more beneficent for long time confinement of a single sign of charge in an ELTRAP device due to its flexibility, large volume, and high standardization [4]. In this numerical study behaviour of electrons in non-neutral plasma is deliberated by using particle-in-cell (PIC) code. The foundational work for plasma simulation was established in the well-known book "Plasma Physics via Computer Simulation" by Birdsall and Langton [5]. In simulation electron plasma is divided into a large number of small computational "particles" their movements are tracked as they interact with the electric and magnetic fields. The fields are calculated by solving the pertinent partial differential equations, taking into account the distribution of charges represented by the particles [6]. The particles are then moved using the calculated fields and the process is repeated over time to obtain a simulation of the electron plasma dynamics [7].

In our research work electron cyclotron resonance heating method is used for the evolution of heating, ionization, and excitation behaviour of the confined electron Plasma by two-dimensional Particle-In-Cell (PIC) code [6]. We ionize the neutral gas atoms by applying (VRF) on one of the electrodes of the electron trap device to separate electrons and ions from neutral particles and gain plasma with a single sign of charge. For axial confinement of electron plasma in an ELTRAP device an electrostatic potential of -100 V is applied on end electrodes and radial confinement is achieved by applying a radial magnetic field. Non-neutral plasma confinement can be accomplished by static electric and magnetic fields [4, 8]. Electron plasma generation and heating impact were studied experimentally by B Paroli for hydrogen background gas by using the Monte Carlo Collision method, and he reported valuable findings for RF-drives (0.1–20) MHz range [9]. The total charge confined in ELTRAP has studied frequency versus dynamical equilibrium in two geometries (realistic and short) traps. When the confinement length of the trap device is half then plasma creation is difficult and the charge confined is half as compared to the long trap. Axial and radial electron losses are balanced by the continuous excitation of residual gas. According to biographer approbation more studies are required to understand the effect of geometry and magnetic field on plasma dynamics [10].

M. Ikram et al reported valuable findings about RF heating phenomena, excitation, and ionization rate by using the Monte-Carlo Collision method about the electron plasma dynamics under hydrogen background gas [11]. The electrostatic simulations were accomplished at different conditions such as various pressures (10^−8^,10^−7^,10^−6^) torr, two different densities (5 ×10^7^ m^-3^, 5 ×10^12^ m^-3^), (1 to 15 MHz) RF drives frequencies and powers (5 and 10 volts) were applied axially at these two positions (C_5_ and C_7_) on the electrostatic stack of the electron trap device. In light of the recommendations provided in the summary further systematic numerical studies were performed by electron cyclotron resonance heating method (ECRH) to know about electron plasma dynamics in terms of axial and radial heating, axial kinetic energy and self-consistent electric field in the realistic trap for hydrogen and helium background gases. Higher heating effects are investigated at low frequency and amplitude in the trap device when the amplitude is applied to cylinder C_8_ as compared to the C_7_ and C_5_ cylinders of the ELTRAP device. Indeed, for the development of electron plasma secondary electrons creation mechanisms plays an important role.

In our numerical simulations same geometry, pressure, and density are chosen for both background gases to compare the confined electron plasma aspects under various conditions, such as for varying amplitudes (1–3.8) V at a constant frequency of 1 GHz RF and also for different RF (0.5–8) GHz at a constant amplitude of 3.8 V applied to C_8_ cylinder of ELTRAP device up to one hundred microsecond’s simulation time. Density and pressure are closely associated with the collision and ionization of background gases. The mathematical relation for calculation of the Brillouin limit of our electron trap device is given below,

$$n_{e}\leq \frac{\varepsilon_{ο}B^{2}}{2m_{e}}=6.1926\times 10^{15}m^{-3}$$ (1)

In all simulation schemes density of the confined gas is taken within the Brillouin limit to decrease the signal-to-noise ratio and losses. In electromagnetic simulations, resonance phenomena occur within the trap device, leading to the formation of electron plasma as a result of matching the applied frequency with the corresponding magnetic field. The most effective way to create single sign charge plasma is through the electron cyclotron resonance heating method because it can operate at low pressure than the Monte Carlo collision method etc. The mathematical relation for cyclotron frequency of electron plasma is given as,

$$\omega_{c}={\left(\frac{e}{m}\right)}_{ele}B$$ (2)

Where e represents charge of an electron, m represents mass and B represents applied magnetic field. By putting the values of these constants and chosen magnetic field we can calculate the cyclotron frequency of the electron plasma. Due to external applied magnetic field electron plasma gyrates within the circle. Mathematically cyclotron frequency and Larmor radius of electron plasma are related as,

$$r_{L}=(\frac{v_{th}}{\omega_{c}})$$ (3)

Where, *v*_*th*_ is thermal velocity, *r*_*L*_ is larmo radius and *ω*_*c*_ is cyclotron frequency of electron plasma. The ECRH method is a more eminent way of providing energy to electrons in the ELTRAP device during cyclotron motion. During cyclotron motion, charged particles are accelerated by varying electric and static magnetic fields to keep them in a spiral path inside the trap device. Electron Plasma excited in the presence of an applied magnetic field satisfies the resonance condition. A small variation in axial motion keeps the total canonical angular momentum conserved as the axial motion alternates between the kinetic and potential states. The confinement of the electron plasma depends on factors such as background gas density, applied potential, and the variation of VRF up to a certain level. The energetic electrons interact with gas atoms and ions present in the ELTRAP device to produce further ionization of neutral particles and the formation of secondary electrons. The background gas density is proportional to ionization and collision rate, by the decrease of pressure electrons accumulation decreases also at high vacuum pressure plasma density is more frequent and higher. The ionization model is deduced from the Saha Boltzmann equation by which we can calculate the relative population of charge state and average ionization of plasma as a function of temperature and density [12]. The noble gases have the economical advantage of generating electron plasma within confinement devices like ELTRAP, due to their higher excitation and ionization rate in trap devices at low power and Radio Frequencies under ultra-high vacuum conditions. These factors play a deep impact on electron plasma losses, collision of gas molecules, space charge perturbation, and limitations of the device [13]. In continuation to understand the underline physics of the influence of two different background gases electromagnetic simulations were performed by electron cyclotron resonance heating method in ELTRAP by using Particle-in-cell code. We have evaluated heating phenomena, excitations, ionizations, and secondary electron productions under hydrogen and helium background gases in realistic trap. In particular electron plasma has the advantage to produce Power in fusion nuclear reactors, growth of microwaves, modifying the surface of a material, positron inhalation, activating the process, production of low-frequency positron beams, sensor fabrication and also in the field of microelectronics [14].

This research work is valuable in engineering such as for studding wave phenomenon in electron positron plasma, fluid dynamics using the traps for electron plasmas; beam Physics, e.g., beam transport systems and coherent radiation devices, for instance, in the free electron lasers, in cyclotron auto resonance masers. The pulsed electron beams can be used to generate coherent x-rays in Thomson back scattering x-ray sources and in the free electron laser.

## 2. Geometry, confinement, and input parameters

The ELTRAP device is used to confine electron plasma by electron cyclotron resonance heating method under two different background gases (Hydrogen and Helium). In this numerical study, Malmberg Penning trap device is used to confine electron plasma computationally. It consists of ten cylinders (C_1_—C_10_) made up of hollow metal each having length of 10 cm with a diameter of 0.09 meter. These cylinders are essential components of the device contributing, in its overall structure and function. The size and shape of the electrodes play a crucial role in confining electron plasma for a long period, which is the primary purpose of the device. Here, power (VRF) is injected into the trapped neutral gas atoms through an electrode C_8_ which acts as an antenna, and as a result of resonance excitation and ionization occurs. Axial confinement is achieved by applying an electrostatic potential of -100 V to the end electrodes. Experimentally the electrostatic stack of ELTRAP is surrounded by a solenoid, who’s axially, highly homogeneous applied magnetic field is used for the radial confinement of the plasma. Thus radially provided magnetic field by the solenoid becomes parallel to the axial direction of the trap. Here, in electromagnetic simulation the radial confinement of electron plasma is achieved by applying radial magnetic field *B*_*Z*_ = *B*(0,0,*B*_1_) tesla are inserted into “control block” of particle in cell code. In our numerical studies, we use Particle-In-Cell code and choose the same geometry and input parameters for both background gases (Hydrogen and Helium) to compare our electromagnetic simulation results with experimental results [6, 7]. The behavior of electron plasma also depends on the vacuum inside the trap device. For long-time confinement of single species (electrons plasma) cylindrical geometry is more suitable and is used in most of the experiments.

The Particle-In-Cell (PIC) method is a popular computational technique, used for confinement of non neutral plasma. It consists of three major blocks; Grid block, Control block, and Load block. The input parameters are inserted into the input text file of PIC code, which consists of these three blocks. Grid block is used to define the geometry shape and size of the ELTRAP device. The value of external magnetic field, electric field and simulation time-steps are inserted into the control block. Also, temperature, density of electron plasma and Maxwell’s distribution of each particle is selected by load block. In configuration space, the PIC code is two dimensional, and in velocity space, it is three dimensional. For each particle or specie a Maxwellian velocity distribution is weighed. The confined particles velocity is computed along radial, azimuthal, and axial direction, while magnetic field is directed along axial direction of trap device as depicted in Fig 1. It incorporates solvers for electrostatic and electromagnetic fields, supporting both Cartesian (x, y) and cylindrical (z, r) geometries. Charge and current densities, alongside the fields, are computed on an orthogonal grid, which may be non-uniform.

In this study, confined gas has a density of 5 x 10^14^ m^-3^ taken within the Brillouin limit and pressure of 10^−5^ torr in a realistic trap. The density is taken within the Brillouin limit to decrease the signal-to-noise ratio and losses during electron plasma formation. In electromagnetic simulations, when the applied frequency varies, the cyclotron frequency and magnetic fields also change, leading to fluctuations in Brillouin’s limit accordingly. The corresponding magnetic field is 0.0357 Tesla for these varying amplitudes 1 V, 1.8 V, and 3.8 V at a constant frequency of 1 GHz. Secondly, amplitude 3.8 V is kept constant for these varying RF 0.5 GHz, 5 GHz, 8 GHz, the corresponding magnetic fields for these applied radio frequencies are presented in Table 1. The size, geometry of the trap device and input parameters were taken with great care to take balance between precision and accuracy.

**Table 1:** Simulation input parameters for the confinement of electron plasma.

Parameters	Standards	Unit
Length of ELTRAP	1	Meter
Radius	0.045	Meter
J	450	Cell
K	40	Cell
Brillouin Limit	6.1926 × 10^15^	Meter^3^
Density	5 × 10^14^	Meter^3^
Np2c	1.76625 × 10^7^	Particles
Pressure	10^−5^	Torr
Cell Size(dx_1_, dx_2_)	0.002222, 0.001122	Meter
Electrostatic Potential	-100	Volt
Magnetic Field for 0.5 GHz	0.017862	Tesla
Magnetic Field for 1 GHz	0.035706	Tesla
Magnetic Field for 5 GHz	0.178620	Tesla
Magnetic Field for 8 GHz	0.285792	Tesla
Simulation Time	(1PS-100μS)	Second
Time Step	2.023 × 10^−12^	Second

The choice between electrostatic or electromagnetic simulations was determined computationally based on the parameters set by the "electrostatic flag," with a value of 1 indicating electrostatic simulations and a value of 0 indicating electromagnetic simulations. When the electrostatic flag is set at 1 then the particle in cell code uses Cartesian coordinates and when it is switched on 0 then the code uses cylindrical coordinates, with X_1_, X_2_, and X_3_ corresponding to z, r, and θ respectively. The electromagnetic simulation length lower coordinates are X_1s_ = 0.00 cm and upper coordinates are X_1f_ = 100 cm along the z-axis, while along the radial direction (r) lower and upper coordinates are represented by X_2s_ = 0.00 cm and X_2f_ = 4.5 cm respectively. The number of cells depends on the device size and geometry. The number of cells that can be distributed in each of the ten cylinders is J = 450 and K = 40 along both axial and radial directions. Super particles are the ratio of actual particles per simulated particles and their accuracy is also affected by no of particles used per unit cell. The mathematical relation for super particles is,

$$Np2c=\frac{n_{e}V}{P_{PC}KJ}$$ (4)

Where *n*_*e*_ represents density of the trapped electrons, volume of the ELTRAP device by V and *p*_*pc*_ represents the number of particles per unit cell. The time step is a way of examining and analyzing the data at specific time interval. To ensure the sustainability and high accuracy of electron plasma simulations, it is better to use a simulation time step that is three orders of magnitude smaller than the cyclotron period. The PIC code, which employs the electron cyclotron resonance heating method, is used to analyze the heating effect, ionization rate, axial kinetic energy, and self-consistent electric field arising due to hydrogen and helium background gases. The schematic diagram for ELTRAP device is shown in Fig 1.

## 3. Electron cyclotron resonance heating in hydrogen and helium background gases

**Fig 2:**
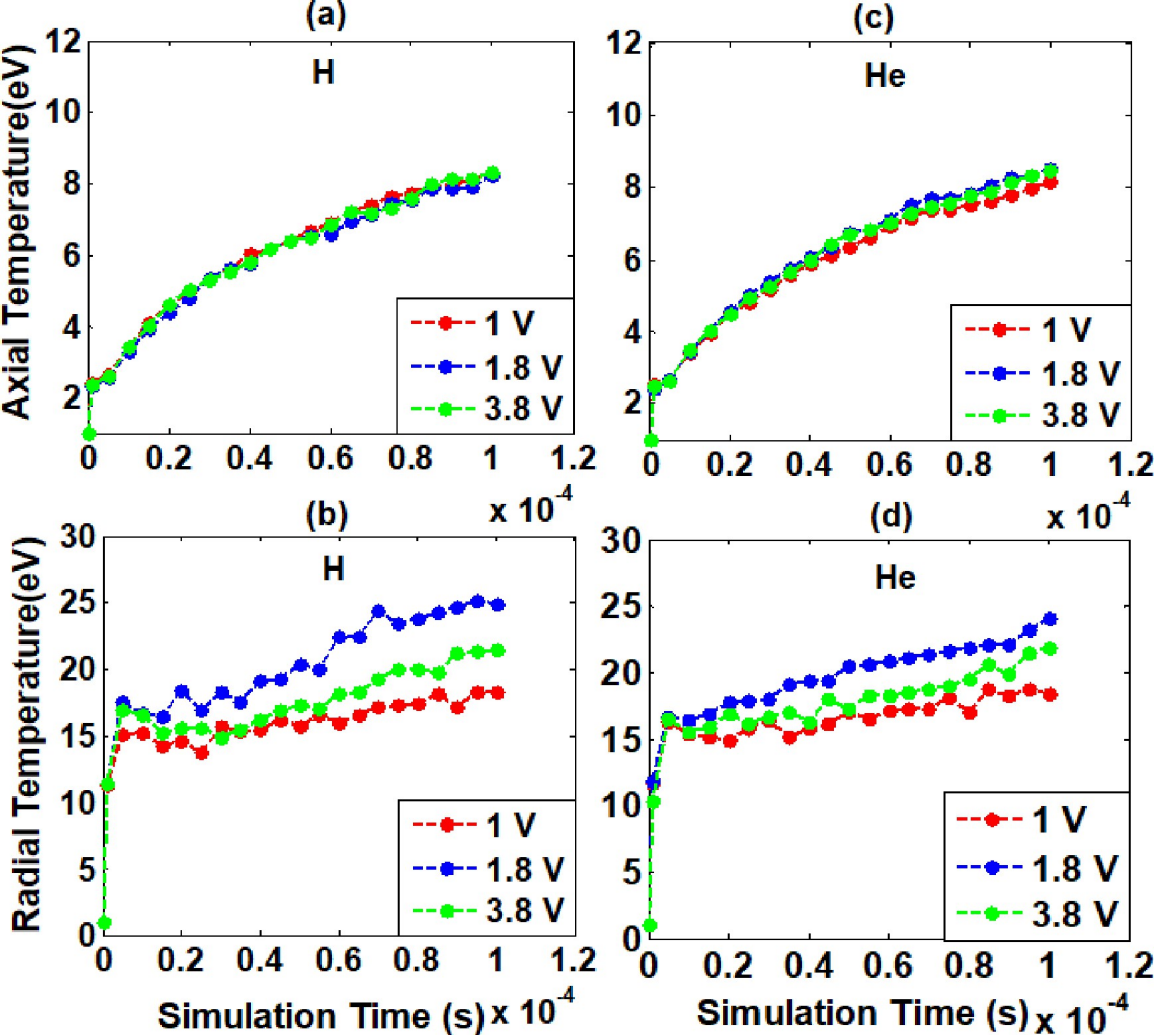
Axial and radial temperatures verses simulation time at background gases Hydrogen (a, b) and Helium (c, d). The simulation performed at applied constant frequency of 1 GHz resonate with magnetic field 0.035706 tesla of different amplitudes for the cases indicated in the legend applied to C_8_ cylinder of ELTRAP.

In ELTRAP device, electron plasma heating is studied by electron cyclotron resonance heating method using Particle-in-cell code. The geometry, density and pressure are kept same for both background gases. Electron plasma was confined axially by applying electrostatic negative potential on end electrodes and for radial confinement magnetic field was applied radially. In electron cyclotron resonance heating mechanism magnetic field varies with applied Radio Frequencies. In this numerical study, to know heating effect at constant frequency of 1 GHz with corresponding magnetic field of 0.035706 tesla used under hydrogen and helium as background gases. The simulation performed at 1 GHz resonance frequency of amplitudes 1 V, 1.8 V and 3.8 V applied to end electrode C_8_ of electrostatic stack of ELTRAP device. The obtained axial temperature are 8.24 eV, 8.23 eV, and 8.30 eV and radial temperature are 18.25 eV, 24.88 eV, 21.45 eV at simulation time of 100 μs under hydrogen background gas as shown in Fig 2(A) and 2(B). Also, the axial and radial temperatures are 8.15 eV, 8.52 eV, 8.46 eV and 18.37 eV, 25.08 eV, 21.87 eV respectively at simulation time of 100 μs under helium background gas as shown in Fig 2(C) and 2(D). Axial temperature increases with increase of RF amplitude in all simulations. But, this increase in temperature along axial direction is very small for both background gases as shown in Fig 2. The reason is their temperature values are less than first ionization energies of hydrogen and helium background gases, i.e. 13.6 eV and 24.58 eV respectively. Due to which high energy particles cannot induce ionization. Consequently, the ionization rate and heating effects remain low along the axial direction of the ELTRAP device.

However, the radial temperature increases as power is raised from 1 V to 1.8 V and decreases with increased power of 3.8 V in the presence of hydrogen background gas. Moreover, at helium backgrounds gas both axial and radial temperatures are found maximum at 1.8 V. Also with increase of RF power from 1.8 V to 3.8 V temperature decreases, but this decrease in temperature along axial direction is small compared to radial direction. The reason of decrease in temperature at 3.8 V is that the energetic particles collide with other residual gas atoms and electrons, as a result their energy and temperature decreased. The radial temperature found higher with respect to axial temperature in both background gases (Hydrogen and Helium) as shown in Fig 2(A)-2(D). In addition, the axial and radial temperatures in helium background gas are found higher than hydrogen gas at low power of 1.8 V and maximum recorded temperature is 8.52 eV and 25.08 eV respectively. Moreover, from analysis of the recorded data, it is concluded that in resonance phenomenon by varying amplitudes effect on excitation, ionization and heating are very low. The recorded temperature results for hydrogen and helium background gases by electron cyclotron resonance heating method are more advanced and productive than results reported by G. Maero for hydrogen background gas [10].

The axial temperature of the electron plasma refers to the temperature of the electrons along the axial direction determined by the direction of the electric field along trap device. It can be used to determine the degree of thermalization of the electrons and to optimize the performance of the trap device. In the context of quantum information processing, the axial temperature of the electron plasma plays a crucial role in determining the coherence time of the trapped electrons. The coherence time depends on the axial temperature because the thermal motion of the electron plasma can cause the dephasing of the quantum states. By measuring and controlling the axial temperature, the coherence time can be maximized, which is essential for realizing scalable quantum information processing. The radial temperature of the electron plasma, on the other hand, refers to the temperature of the electrons in the direction plane perpendicular to the electric field. By measuring the radial temperature, the energy transfer rate can be estimated, which is important for understanding electron plasma dynamics. The physical significance of measuring the axial and radial temperature lies in the fact that it provides information about electron plasma heating impacts on the performance of the trap device. In particular, the axial and radial temperatures of the electron plasma provide valuable information about behavior of the trapped electrons, their confinement and heating effect produced in the ELTRAP device.

**Fig 3:**
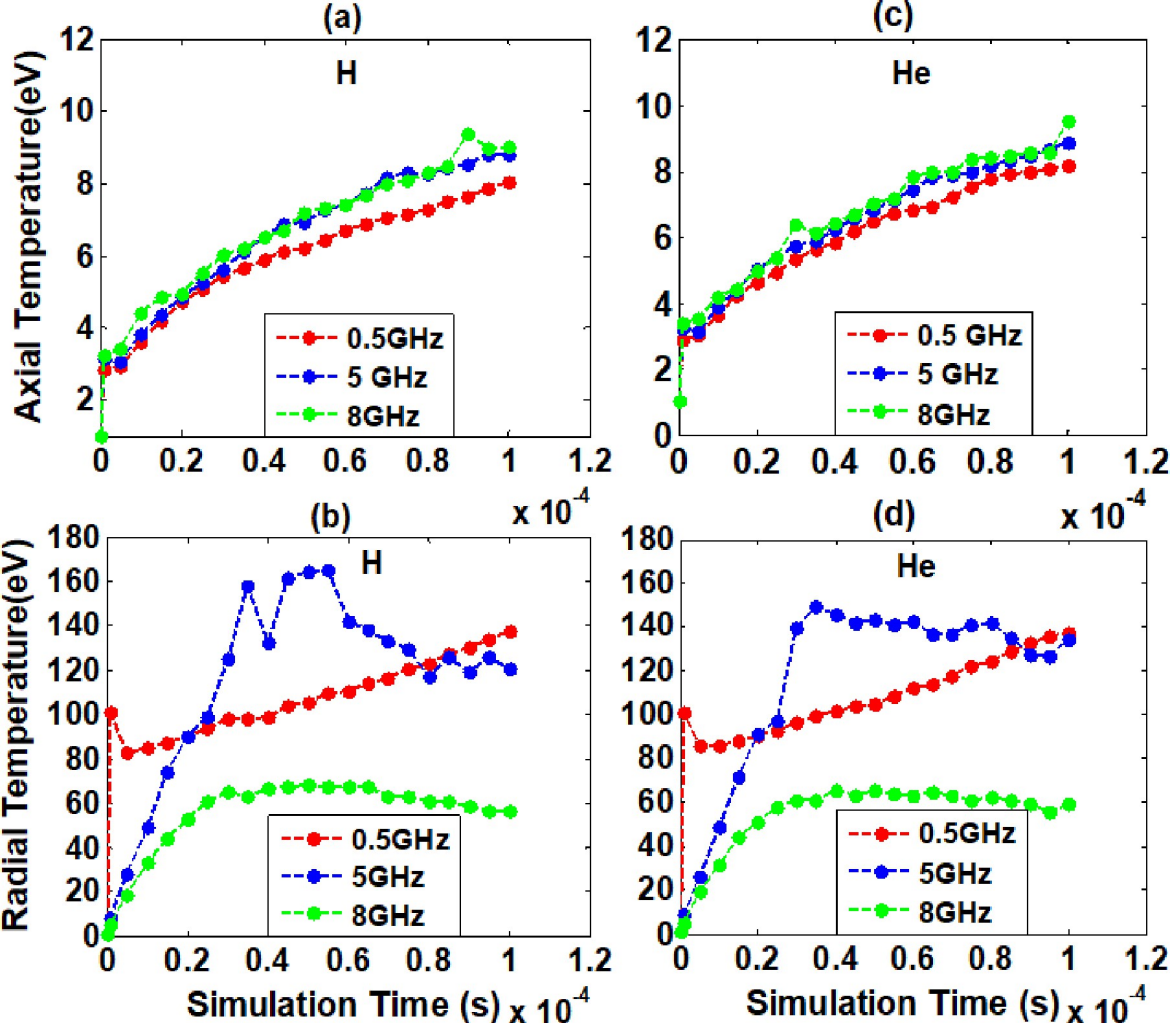
Axial and radial temperatures verses simulation time at background gases Hydrogen (a, b) and Helium (c, d). The simulation performed at constant amplitude of 3.8 V for different radio frequencies indicated in legend matched with corresponding magnetic fields are applied to C_8_ cylinder of ELTRAP.

The electromagnetic simulation was performed by using two-dimensional Particle-In-Cell code to evaluate ionization and excitation of electron plasma at constant amplitude of 3.8 V with three different frequencies. In electron cyclotron resonance method, radio frequencies 0.5 GHz, 5 GHz, and 8 GHz corresponding magnetic fields are 0.017862 Tesla, 0.17862 Tesla and 0.285792 Tesla respectively at Hydrogen and helium background gases. The ionization rate and heating effects increased continuously with the continuous application of RF excitation through C_8_ cylinder of the electromagnetic stack. The obtained axial temperature at simulation time of 100 μs are 8.03 eV, 8.81 eV, and 9.04 eV, and radial temperatures are 137.02 eV, 170.41 eV, 56.54 eV under hydrogen background gas as shown in Fig 3(A) and 3(B). Also, the axial and radial temperatures at simulation time of 100 μs under helium background gas are 8.18 eV, 8.87 eV, 9.54 eV and 137.02 eV, 150.08 eV, 59.62 eV respectively as shown in Fig 3(C) and 3(D). It is observed from graph peaks that axial temperature increased by increasing frequency for both background gases, but radial temperature is found maximum at 5 GHz frequency. The radial temperature of confined electron plasma is found higher in hydrogen background gas compared to helium gas and recorded peak value is 170.41 eV at simulation time of 50 μs as shown in Fig 3. The radial temperature starts decreasing further onward from 50 μs simulation time, because these energetic particles collide with other charged and neutral particles, causing them to lose energy. As a result, average radial temperature decreases, and its behaviour is observed to be non-linear. Moreover, the radial temperature has higher peak at 5 GHz compared to other RF for both background gases. This may be due to resonance and bouncing effect in trapping length between C_2_ to C_9_ electrodes of ELTRAP, and decline of radial temperature (eV) may be due to a higher loss of energetic confined electrons. Additionally, from simulation results we can conclude that the heating phenomenon is found more dynamic and higher at varying radio frequencies than varying amplitudes. Electrons with energy significantly exceeding the first ionization energies of residual gas atoms will make a more substantial contribution to excitation and ionization processes.

The physical significance of axial and radial temperatures in ELTRAP is that they provide valuable information about the properties and behaviour of the trapped electron plasma. The axial temperature provides information about the thermal equilibrium and confinement properties, while the radial temperature provides information about the heating mechanisms of the trap. The charged particles (electrons, ions) have three different types of motions axial, magnetron and cyclotron in ELTRAP device. In resonance cyclotron motion is responsible for heating electrons, which is completely kinetic, while magnetron motion is entirely potential dependent. The radial positions of confined charged particles is described by the equation as,

$$p_{\theta }=\frac{qB}{2c}\sum_{i=1}^{n}r_{i}^{2}$$ (5)

It also conserves the mean square radius of electron plasma. In case of confined electron plasma, the constraints are replaced by $\sum r_{i}^{2}=$ constant so that electrons will gyrate along trap radius. The gyration of electrons is too much high along radial direction, due to which heating effect was found higher along radial direction. Accurate measurements and control of these temperature effects are essential for optimizing the performance of the trap device. The axial and radial heating at constant amplitude with varying RF are as shown in Fig 3.

## 4. Kinetic energy of confined electrons in hydrogen and helium background gases

**Fig 4:**
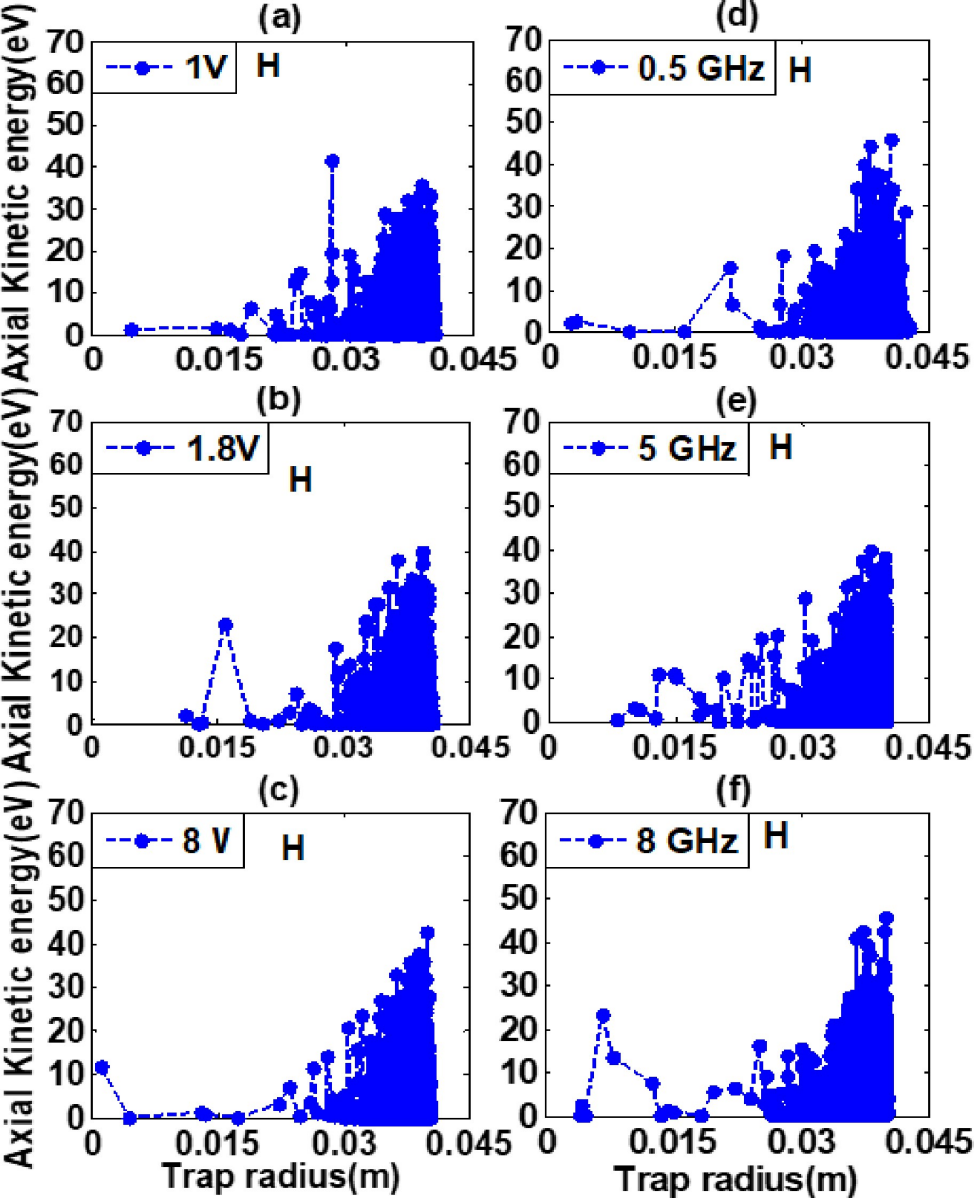
Axial kinetic energy (eV) verses trap radius (m) graphs for hydrogen background gas having density 5×10^-14^m^-3^ at constant RF of 1 GHz for these varying amplitudes as well as for constant amplitude of 3.8 V for these varying RF are shown in legend of Fig 4(A)–4(F) for simulation time of 100 μs and powered to C_8_ cylinder of realistic trap.

**Fig 5:**
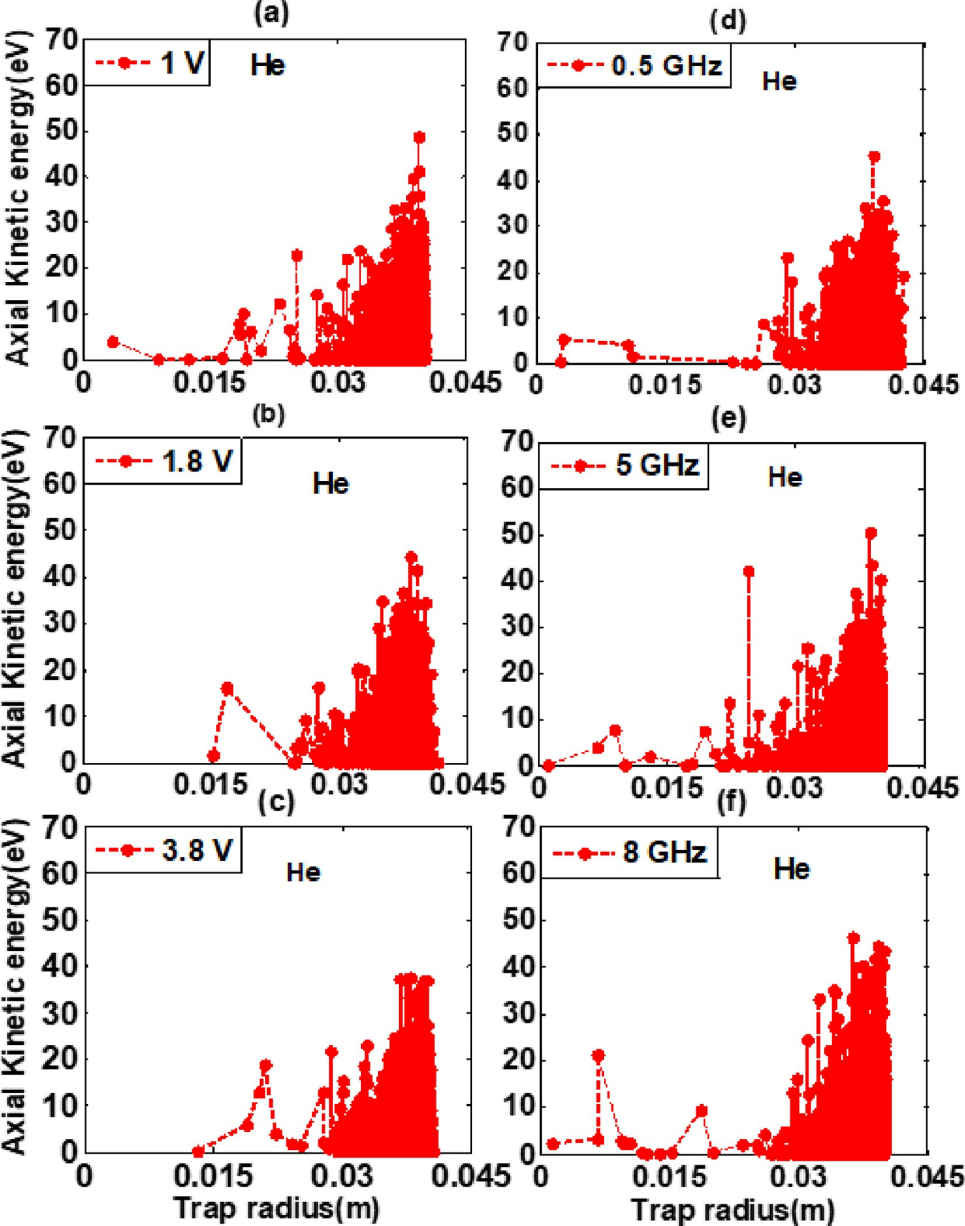
Axial kinetic energy (eV) verses trap radius (m) graphs for helium background gas having density 5×10^-14^m^-3^ at constant RF of 1 GHz for these varying amplitudes as well as for constant amplitude of 3.8 V for these varying RF are shown in legend of Fig 5(A)–5(F) for simulation time of 100 μs and powered to C_8_ cylinder of realistic trap.

The axial kinetic energy (eV) of confined electron plasma is studied in realistic trap by using two-dimensional particle-in-cell (PIC) code for hydrogen and helium background gases. The geometry and input parameters for both background gases are same as discussed in section 2. It was measured in electron volts (eV) and simulation was run for a period of (1Ps-100μs). The recorded peaks for hydrogen and helium background gases at constant RF of 1 GHz at these varying amplitudes 1 V, 1.8 V, and 3.8 V are 41.53 eV, 39.84 eV, 42.58 eV, and 48.54 eV, 44.26 eV, 37.63 eV respectively as shown in Fig 4. The recorded data shows that axial kinetic energy along the trap radius has a maximum value at low power of 1 V than other electric powers for helium background gas. Also, axial kinetic energy in helium background gas was higher as compared to hydrogen background gas. Similarly, at constant amplitude of 3.8 V for these varying frequencies 0.5 GHz, 5 GHz, and 8 GHz the recorded peaks are 46.02 eV, 40.09 eV, 45.91 eV, and 45.55 eV, 50.57 eV, 46.44 eV respectively as shown in Fig 5. It is concluded from graphs analysis that the axial kinetic energy of hydrogen background gas has a maximum value at 0.5 GHz, while for helium background gas maximum value was obtained at 5 GHz than other excitation frequencies. Moreover, it is also concluded that axial kinetic energy of helium background gas was higher as compared to hydrogen background gas and recorded peak value is 50.57 eV. Additionally, the electron heating is initially stronger close to the wall of the ELTRAP in the range of 0.030 meter to 0.040 meter and gains energy radially at the simulation time of 100 micro second. Due to the high electric field created by the excited electrode (C_8_), a higher heating effect is observed near the walls of the trap device. During the simulation, the heating effect extends from the VRF plugging position toward the centre of the trap. The collision between energetic electrons and neutral particles causes the energy of some excited electrons to decrease [15]. As a result, the average radial profiles of axial kinetic energy decrease towards the center of the electron trap device.

The electrons are localized near to wall having energy greater than first ionization energies of hydrogen and helium are able to ionize the residual gas atoms better than the electrons located close to the central part of the trap. The first ionization energies of hydrogen and helium background gases are 13.6 eV and 24.58 eV respectively. Those charged particles having energy greater than first ionization energies are able to cause further ionization and secondary electron production. For excitation and ionization rate helium background gas required more energy as compared to the hydrogen background gas. It is due to resonance effect of electron cyclotron resonance heating method that energetic excited electrons and slow moving charged particles resonate equally. It is independent of charged particles energies. The particles having high energy gyrate with large Larmor radius and those having low energy gyrate with small Larmor radius. Thus electrons which carry high energy are pushed towards wall of ELTRAP device. From this, we can conclude that most of the ionization takes place in this region. The axial kinetic energy along the trap radius is an important aspect of plasma behaviour that affects the electron plasma formation in ELTRAP device. It was found from graph analyses that at 5 GHz RF more ionization occurred due to which heating effects were higher in both background gases. Electron plasma temperature and energy effects for both background gases (Hydrogen and Helium) due to the electron cyclotron resonance heating method are more advance and reliable as compared to results reported by M. Ikram et al for hydrogen background gas by using Monte-Carlo collision method [11].

The axial kinetic energy of the electron plasma in ELTRAP is a key parameter that describes the energy along the trap radius. It is related to electrons thermal energy through the equipartition theorem, which states that the energy of each degree of freedom of a particle in thermal equilibrium is equal to $\frac{KT}{2}$, where K represents Boltzmann constant and T represents temperature. Therefore, by measuring the temperature of the electron plasma, we can obtain information about kinetic energy of the electrons along the axial direction. The confinement of the electrons along the axial direction depends on the balance between the magnetic field strength and the axial kinetic energy of the electrons. If the axial kinetic energy of the electrons is too high, they can escape from the confining magnetic field, leading to a loss of confinement and a reduction in the plasma density. Therefore, controlling the axial kinetic energy of the electrons is important for maintaining highly confined electron plasma in ELTRAP. The stability of the electron plasma depends on the balance between the magnetic field strength, the plasma density, and the axial kinetic energy of the electrons. Instabilities can arise due to the interaction between the charged particles and the magnetic field, which can cause the electron plasma to lose its confinement and become unstable. If the axial kinetic energy of the electrons is too high, it can destabilize the plasma and lead to instability. Therefore, controlling the axial kinetic energy of the electrons is important for maintaining stable electron plasma in ELTRAP.

## 5. Self-consistent radial electric field

Electron plasma generated in ELTRAP device, creates an electric field due to these charged particles. Coulomb force is responsible for the creation of an electric field between these charged particles. The self-consistent electric field is studied along radial direction of ELTRAP device by using electron cyclotron resonance heating method at varying amplitudes and radio frequencies. The self-consistent electric field of charge particles (electrons) in ELTRAP device is studied by electron cyclotron resonance heating method of Particle-In-Cell code. Self-consistent electric field of these confined electrons responds to the external applied fields. The confined electron resonates with matching of applied radio frequencies with corresponding magnetic fields. Thus, the charge particles gain energy and gyrate along the magnetic field lines. The electric field is the negative gradient of potential and can be written as,

$$\vec{E}=-\nabla \varphi$$ (6)

The potential is usually proportional to the distance between the end cylinders with a negative potential indicating axial confinement of electron plasma. The laplacian equation [16] can be written in speherical polar coordinates (*r*,*θ*,*ϕ*) as,

$$\nabla^{2}\varphi (r,\theta ,\phi )=\frac{1}{r^{2}\sin\theta }[\sin\theta \frac{\partial }{\partial r}(r^{2}\frac{\partial \phi }{\partial r})]+\frac{1}{r^{2}\sin\theta }\frac{\partial }{\partial \theta }(\sin\theta \frac{\partial \phi }{\partial \theta })+\frac{1}{r\sin^{2}\theta }(\frac{\partial^{2}\phi }{\partial \phi^{2}})=0$$ (7)

As heating effects are found higher along radial direction compare to axial direction in ELTRAP device. The electric field is smaller along axial and azimuthal directions as result take into acount the radial electric field (volt/meter). The radial part of laplacian is given by,

$$\nabla^{2}\varphi (r,\theta ,\phi )=\frac{1}{r^{2}}\frac{\partial }{\partial r}(\frac{\partial \varphi }{\partial r})$$ (8)

Maxwell’s equations and Lorentz force are used to solve the electromagnetic field generated by electron cyclotron resonance heating method by using particle in cell code in two dimensions. The mathematical relation for Lorentz force is given by,

$$\vec{F}=q(\vec{E}+\vec{v}\times \vec{B})$$ (9)

Where particle’s charge is denoted by q, velocity is symbolized by $\vec{v}$, electric field by $\vec{E}$, and magnetic field by $\vec{B}$. The last part in the Lorentz force equation has disappeared because plasma is sensitive to self-induced magnetic fields rather than external magnetic fields. Equation of motion used to describe the position, velocity, and acceleration of each micro charged particle is given by,

$$m\frac{d\vec{v}}{dt}=q\vec{E}$$ (10)

Here, m represents mass, and $\vec{v}$ represents velocity of charged particle. Non-neutral plasma is comprised of charged particles, and the PIC code is utilized to analyze both the current and charge density. The charged particles (electrons) interact with each other as well as with fields, and this is what allows for the examination of the current densities [17]. Now, beginning with the charge and current densities given to the grid points, we used *ρ* and J as input to compute the electric and magnetic fields. Maxwell’s equations enable the computation of external fields through the use of Newton’s law, while the behavior of particles in these fields is monitored by the Lorentz force [18]. The data is collected in three dimensions (*r*,*θ*,*ϕ*) to observe heating phenomena, axial kinetic energy and self-consistent electric field due to these charged particles.

The divergence of electric field is,

$$\nabla \cdot \vec{E}=\frac{\rho }{\varepsilon_{\circ }}$$ (11)

Poisson’s equation is deduced from mathematical relation expressed in Eqs 1 and 6, i.e. fundamental equation in electromagnetism that relates the charge density to the electric field. In order to compute the electric field using the Poisson equation, it is necessary to first determine the charge density at any given point within space. Poison’s equation is solved using mathematical techniques such as partial differential equations and boundary conditions, which take into account the geometry and symmetry of the charge distribution. PIC code can solve equations of motions for micro charge particles, and Maxwell equations for electromagnetic fields [19] are given by,

$$\nabla \times \vec{E}=\frac{-\partial \vec{B}}{\partial t}$$ (12)

$$\nabla \cdot \vec{B}=0$$ (13)

$$\nabla \times \vec{B}=\mu_{\circ }\varepsilon_{\circ }\frac{\partial \vec{E}}{\partial t}$$ (14)

For charge conservation,

$$\frac{\partial \rho }{\partial E}+\Delta \cdot \vec{J}=0$$ (15)

Our investigation focused on the self-consistent radial electric field resulting from confined electron plasma along the trap radius within a realistic trap. At constant RF of 1GHz for these varying amplitudes (1 V, 1.8 V, and 3.8 V) as well as for constant amplitude of 3.8 V for these varying frequencies (0.5 GHz, 5 GHz, and 8 GHz) the minimum and maximum values of a self-electric field are tabulated in Table 2. It is recorded from graphs analysis that by increasing amplitude radial electric field increases for both background gases. The maximum value of the radial electric field at amplitude of 3.8 V for hydrogen gas is 1.007×10^6^ Vm^-1^ and for helium background gas is 1.033×10^6^ Vm^-1^. While increasing frequency from 0.5 GHz to 5 GHz self-consistent radial electric field increases and on further higher RF of 8 GHz, it starts decreasing. At 5 GHz the maximum recorded value for hydrogen background gas is 1.096×10^6^ Vm^-1^ and for helium background gas 1.097×10^6^ Vm^-1^. From these graphs analysis, we reach on this conclusion that electric fields created by varying frequencies are higher than varying amplitudes.

**Table 2:** The Self-consistent radial electric field at various powers and radio frequencies for hydrogen and helium background gases.

Self-consistent radial electric fields at various powers and Radio Frquencies					
				H	He
**1 V**	x-min	0.000 m	y-min	-5.349 × 10^5^ Vm^-1^	-5.401 × 10^5^ Vm^-1^
	x-max	0.045 m	y-max	6.821 × 10^5^ Vm^-1^	7.040 × 10^5^ Vm^-1^
**1.8 V**	x-min	0.000 m	y-min	-4.711 × 10^5^ Vm^-1^	-4.697 × 10^5^ Vm^-1^
	x-max	0.045 m	y-max	8.095 × 10^5^ Vm^-1^	7.737 × 10^5^ Vm^-1^
**3.8 V**	x-min	0.000 m	y-min	-5.751 × 10^5^ Vm^-1^	-5.694 × 10^5^ Vm^-1^
	x-max	0.045 m	y-max	1.007 × 10^6^ Vm^-1^	1.033 × 10^6^ Vm^-1^
**0.5 GHz**	x-min	0.000 m	y-min	-5.966 × 10^5^ Vm^-1^	-5.818 × 10^5^ Vm^-1^
	x-max	0.045 m	y-max	8.081 × 10^5^ Vm^-1^	8.370 × 10^5^ Vm^-1^
**5 GHz**	x-min	0.000 m	y-min	-5.531 × 10^5^ Vm^-1^	-6.446 × 10^5^ Vm^-1^
	x-max	0.045 m	y-max	1.096 × 10^6^ Vm^-1^	1.094 × 10^6^ Vm^-1^
**8 GHz**	x-min	0.000 m	y-min	-7.956 × 10^5^ Vm^-1^	-7.384 × 10^5^ Vm^-1^
	x-max	0.045 m	y-max	4.443 × 10^5^ Vm^-1^	8.826 × 10^5^ Vm^-1^

The minimum and maximum values of the self-consistent electric fields are recorded between (0–0.045) meter along radius of ELTRAP device for both background gases. When electron plasma is formed in ELTRAP, it generates an electric field due to the presence of charged particles. The self-consistent radial electric field exhibits higher values in the range of 0.025 meters to 0.045 meters along the trap radius as compared to other sites inside the trap device. The electric field, which is created by the confined electron plasma, is largely restricted to the vicinity of the trap wall.

**Fig 6:**
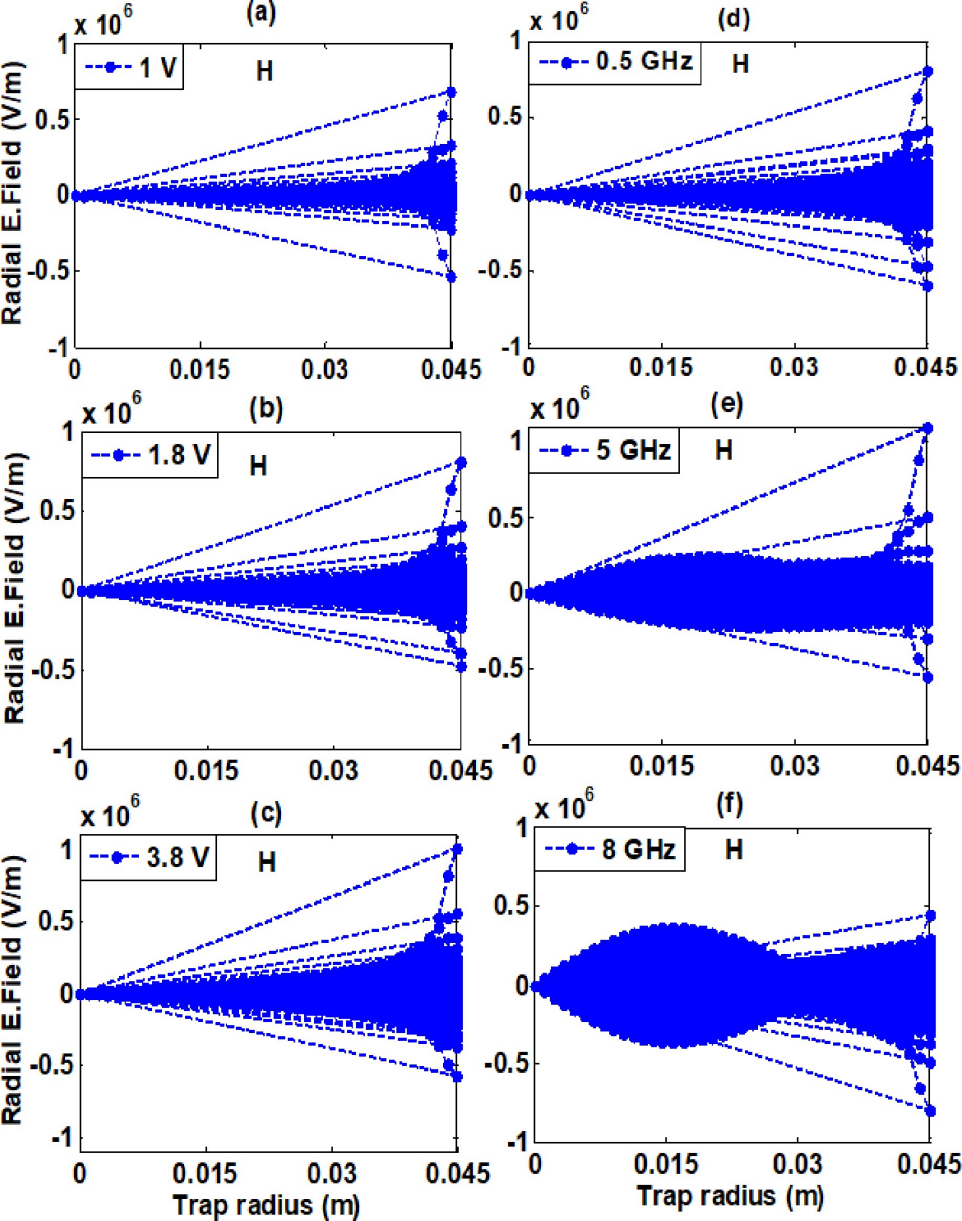
Radial electric field along trap radius at constant RF of 1 GHz for these varying amplitudes as well as for constant amplitude of 3.8 V for these varying RF shown in legend for hydrogen background gas for simulation time of 100 μs having background gas density 5×10^−14^ m^-3^ and powered to C_8_ cylinder in realistic trap.

**Fig 7:**
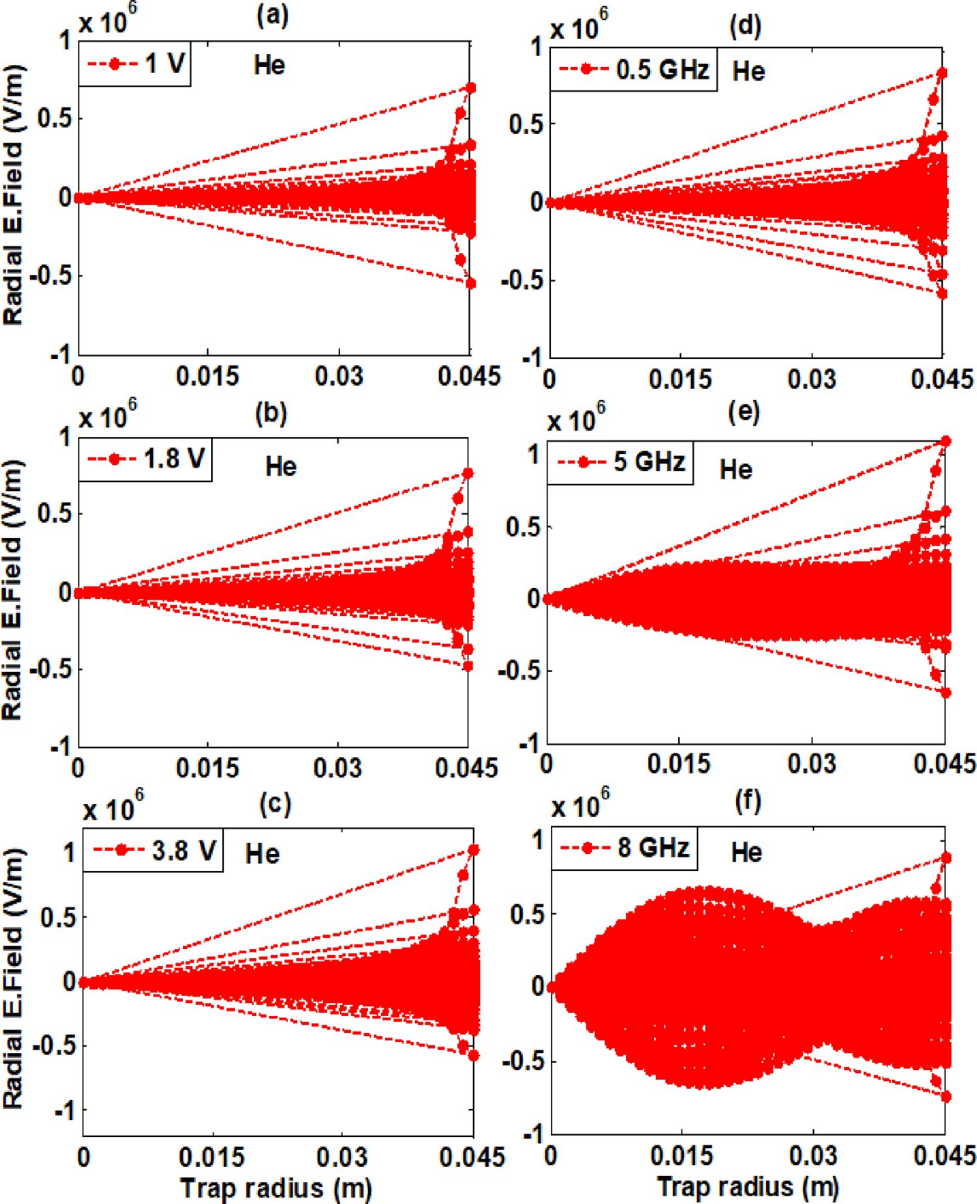
Radial electric field along trap radius at constant RF of 1 GHz for these varying amplitudes as well as for constant amplitude of 3.8 V for these varying RF shown in legend for hydrogen background gas for simulation time of 100 μs having background gas density 5×10^−14^ m^-3^ and powered to C_8_ cylinder in realistic trap.

The self-consistent radial electric field arises as a result of negatively charged particles (electrons) within the plasma. The physical significance of this electric field is that it plays a crucial role in the behavior of the electrons within the plasma. For example, the electric field can influence the trajectory of the electrons as they move through the trap. Specifically, external magnetic field can cause the electrons to spiral around the axis of the trap, which helps to confine them within the ELTRAP device. The electric field can also affect the energy of the electrons within the plasma. As the electrons move through the field, they can gain or lose energy depending on their direction of motion. This energy exchange can lead to the heating or cooling of the electron plasma, which in turn can affect its overall behavior. Overall, the electric field created during electron plasma formation in an ELTRAP shapes the behavior of the electron plasma and its interactions with the surrounding environment. The self-consistent radial electric field graphs for hydrogen and helium background gases are shown in Figs 6 and 7 respectively.

## 6. Conclusion

Electron plasma is generated in the ELTRAP device by using electron cyclotron resonance heating method. The heating effect, axial kinetic energy, and self-consistent electric field effects of trapped electron plasmas were analyzed using a two-dimensional Particle-In-Cell code for both hydrogen and helium background gases. It is concluded from graphs analysis that axial temperature (eV) of electron plasma has a maximum value at low power of 1.8 V and radial temperatures peaks were observed at 5 GHz for both background gases. Radial temperature exhibits a greater increase than axial temperature, and the heating effects were found to be more significant when varying radio frequencies compared to varying amplitudes. Moreover, radial temperature behavior was to be non-linear at 50 μs simulation time, and maximum recorded value for hydrogen was 170.41 eV. The non-linear behavior can be attributed due to energetic electrons collision with other electrons and gas atoms, which leads to a decrease in their energy.

In start, axial kinetic energy of the confined electron plasma along the trap radius was uniform; Later on, with increasing simulation time trapped electrons are driven toward the wall of the trap device, and the larger heating impact were observed in the region 0.025 m to 0.04 m due to higher ionization and collision. As high-energy electrons move toward the walls of the trap device, with increasing Larmor radius, the energetically excited electrons supply the threshold energy to the background gas and free electrons. Consequently, this leads to an increase in the ionization rate and the production of secondary electrons. At a frequency of 5 GHz and a power of 1 V, the outcomes for axial kinetic energy in helium were observed to be more advanced than in hydrogen gas. The self-consistent electric fields were found higher in helium background gas compared to hydrogen background gas and its maximum recorded values at 3.8 V amplitude and 5 GHz RF are 1.033×10^6^ Vm^-1^ and 1.097×10^6^ Vm^-1^ respectively. In light of the above discussion, it is concluded that both background gases are more consistent for producing ionization and heating effects.

## Abbreviations

ω_c_: cyclotron frequency of electron plasma and its unit is hertz
r_L_: Larmo radius of electron plasma and its unit is meter
eV: electron volt, the unit of energy and 1*eV* = 1.602×10^−19^ Joule
*Np*2*C*: Number of super particles used in simulation
KT: Boltzmann constant and temperature product, where *K* = 1.380649×10^−23^*JK*^−1^
